# Antimicrobial, Cytotoxic and Antioxidant Activities and Determination of the Total Tannin Content of Bark Extracts *Endopleura uchi*

**DOI:** 10.3390/ijms12042757

**Published:** 2011-04-21

**Authors:** Flávio A. S. Politi, João C. P. de Mello, Ketylin F. Migliato, Andréa L. A. Nepomuceno, Raquel R. D. Moreira, Rosemeire C. L. R. Pietro

**Affiliations:** 1 Department of Drugs and Medicines, School of Pharmaceutical Sciences of Araraquara, UNESP-São Paulo State University, Rodovia Araraquara-Jaú, Km 01, 14801-902, CP 502 Araraquara (SP), Brazil; E-Mails: politif@fcfar.unesp.br (F.A.S.P.); migliato@fcfar.unesp.br (K.F.M.); 2 Department of Pharmacy, Universidade Estadual de Maringá. Avenida Colombo, 5970, 87020-900 Maringá (PR), Brazil; E-Mail: mello@uem.br (J.C.P.M.); 3 Department of Natural Active Principles and Toxicology, School of Pharmaceutical Sciences of Araraquara, UNESP-São Paulo State University, Rodovia Araraquara-Jaú, Km 01, 14801-902, CP 502 Araraquara (SP), Brazil; E-Mails: alanepomuceno@hotmail.com (A.L.A.N.); moreirar@fcfar.unesp.br (R.R.D.M.)

**Keywords:** *Endopleura uchi*, minimum inhibitory concentration, IC_50_, tannins

## Abstract

*Endopleura uchi* is a typical Amazonian tree and its bark is popularly employed in the preparation of teas against myomas, arthritis, influenza, diarrhea and cancer. In this study, the antioxidant activity, cytotoxicity and antimicrobial activity of five different extracts of the bark, selected by their total tannin content, were assessed. The potential antioxidant activity of the extracts was determined by 2.2-diphenyl-1-picrylhydrazyl radical scavenging assay and the values found were very similar among the extracts and to the standards antioxidants used in the tests. Cytotoxicity analysis in mammalian cells indicated that all the tested extracts exhibited IC_50_ values higher than the highest concentration used, showing that they do not present a risk when consumed under these conditions. Extract tested against five bacterial strains and one yeast strain did not show satisfactory growth inhibitory activity, and even the extracts that showed some antimicrobial activity were not effective at any dilution to determine the minimum inhibitory concentration. The results may serve as a reference for subsequent works, since such reference values described in the literature for the bark of *E. uchi*.

## 1. Introduction

Nowadays, in spite of great developments in organic synthesis and new biotechnological processes, a notable increase in phytotherapeutic practice can be observed. Approximately 25% of the medicines prescribed in the industrialized countries originate from plants and about 120 compounds of natural origin, obtained from approximately 90 species of plants, are used in modern therapy [1]. Furthermore, natural products are involved in the development of about 44% of all new drugs [1]. In Brazil, approximately 80,000 species of plants are described, offering a wide range of raw material for the discovery of new drugs [1]. Clearly, given this enormous variety of species, this potential source of new drugs is far from completely explored and only 17% of this group of plants has been the focus of systematic studies in search of biological compounds [2]. The World Health Organization (WHO) estimates that 65–80% of the people in developing countries use traditional medicine for primary health care and 85% of that involves the use of plant extracts. The WHO recommends research into the use of the local flora for therapeutic purposes, with the intention of reducing the number of people excluded from effective therapy in the government health systems, which could constitute an economically viable alternative treatment of several diseases, especially in developing countries [3,4].

The abusive and indiscriminate use of antimicrobial compounds over many years is the main factor responsible for the appearance of the phenomenon of bacterial resistance to such compounds [5]. Several alternatives have been suggested to solve this problem such as the search for new antimicrobials in plant species [6,7]. Some plants synthesize substances to defend themselves when attacked by bacteria, fungi, parasites, viruses or other agents. These compounds are products of their secondary metabolism and of particular interest are those with antimicrobial properties such as: terpenoids (monoterpenes, sesquiterpenes, diterpenes and saponins), phenolics (simple phenols, tannins, dibenzofurans and flavonoids), nitrogenated compounds (alkaloids, cyclic peptides and glycosides), coumarin and camphor [8–11].

*Endopleura uchi* (Huber) Cuatrec. (Humiriaceae) is a native tree of the Brazilian Amazon rainforest and is found scattered over the whole Amazonian Basin. The family Humiriaceae was described by Antoine Laurent de Jussieu and includes 50 species classified in eight genera, spread in tropical areas of America, and one specie in tropical West Africa. The plant is known in the Amazon as “*uchi*”, “uxi-amarelo”, “cumatê”, “axuá”, “pururu”, “uxi-liso”, “uxi-ordinário” or “uchi-pucu” [12,13]. The erect trees have pale gray bark and reach between 25 and 30 meters in height, with a stem diameter over one meter. The bark is widely commercialized at fairs, markets and even drugstores, being prescribed in the form of tea, for arthritis, cholesterol, diabetes, diarrhea treatments and as an anti-inflammatory [14]. The phytochemical screening of the bark revealed the predominance of tannins, coumarins and saponins as the main classes of secondary metabolites [15]. In previous work, Luna *et al.* (2000) [16] isolated, from crude ethanolic extracts of the bark, two coumarins (bergenin and dimethyl bergenin) and two pentacyclic triterpenoids of the oleanane series (maslinic acid and methyl maslinate).

In this study we determined total tannin contents and investigated the antimicrobial and cytotoxic activities of various extracts of powdered bark of *E. uchi*.

## 2. Results and Discussion

**Figure 1.**
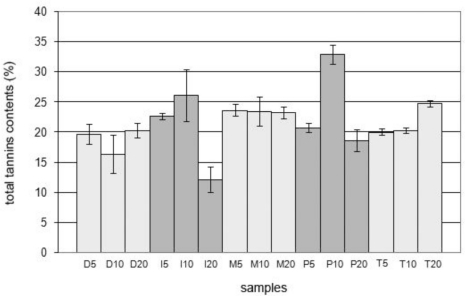
Total tannins contents of extracts of *E. uchi* (Huber) Cuatrec. (Humiriaceae). D_5_: 5% decoction (w/v); D_10_: 10% decoction (w/v); D_20_: 20% decoction (w/v); I_5_: 5% infusion (w/v); I_10_: 10% infusion (w/v); I_20_: 20% infusion (w/v); M_5_: 5% maceration (w/v); M_10_: 10% maceration (w/v); M_20_: 20% maceration (w/v); P_5_: 5% percolation (w/v); P_10_: 10% percolation (w/v), P_20_: 20% percolation (w/v); T_5_: 5% turboextraction (w/v); T_10_: 10% turboextraction (w/v); T_20_: 20% turboextraction (w/v).

Considering that the aim of the present study was to evaluate the antibacterial effect of powdered bark extracts of *E. uchi* and given the antimicrobial activity described for the tannins [17–22], it was important to estimate the contents of this group of substances in the extracts (Table 1 and Figure 1).

Thus, the extracts were selected according to their total tannins contents, to be subjected to the biological tests. The 5% extract with the highest total tannins content was prepared by maceration (23.62% ± 1.0); the 10% extract with highest total tannins content was prepared by percolation (32.85% ± 1.62), this being the extract with the overall highest total tannins content, and the 20% extract with highest total tannins content was prepared by turboextraction (24.77% ± 2.54). Besides these three extracts, the 10% infusion (26.06% ± 4.27) and the 20% decoction (20.27% ± 1.16) were included in the biological tests because popular use is almost totally based on the consumption of teas. It was found that for some extraction processes (decoction and maceration) the total tannins content in the 5% extracts was very close to that in the 20% extracts (*H*_0_ accepted, *P* < 0.05). This makes the 5% extract attractive from the economic point of view, because smaller amounts of plant material are consumed. The values of total tannins (TT) found in the extracts of powdered barks of *E. uchi*, all around 21%, compare favorably with the values found by Yamaguti-Sasaki *et al*. (2007) [23], who used 5% aqueous extract (16.16% ± 0.44), crude acetone:water extract (31.15% ± 1.46) and semi-purified fractions (30.05% ± 0.54; 17.09% ± 0.52) of the seeds of *Paullinia cupana* H. B. K. var *sorbilis* (Mart.) Ducke.

The results of the antimicrobial activity tests are presented in Table 2. The control solution (DMSO:BHI) did not produce inhibition haloes against the microorganisms studied, indicating that this solvent does not interfere in the antimicrobial activity results for the extracts. In the disk-diffusion test in agar, with either templates or filter paper discs; no significant bacterial growth inhibition was observed, except a small activity in the extracts against *S. aureus* and activity of the 10% infusion extract against *C. albicans*. It should be noted that since the tannins form complexes with proteins [24–26], it is possible that local precipitation occurred, impeding the tannins from diffusing in the culture medium, and thus masking their real activity, despite the presence of Tween 80^®^. In general, plant extracts contain low concentrations of highly active compounds and a great number of other compounds that may have promising activities, but which need an appropriately sensitive test to be detected [27]. It is also possible that yet other substances exist in the extracts that interfere with the real antimicrobial potential of the tannins. Thus, it would be very interesting to fractionate the extracts, isolate the compounds and obtain a more accurate assessment.

The minimum inhibitory concentration (MIC) was determined for the strain *S. aureus*. There was bacterial growth in the wells selected as positive growth control and solvent control (DMSO) and no bacterial growth in the wells that did not receive the inoculums (negative controls), indicating the sterility of the culture medium and of the extracts. The antibiotic was shown to be effective (MIC of 0.78 μg/mL), but the extracts showed no activity at any of the dilutions.

In the cytotoxicity tests with fibroblast cells, none of the tested extracts were shown to be toxic, and all the cell survival values were 100%. Thus, the IC_50_ of all the extracts was higher than the highest tested concentration (0.2 mg/mL).

DPPH is a stable free radical that interacts with antioxidant substances, which transfer electrons or atoms of hydrogen to DPPH, neutralizing (“scavenging”) the free radical. This process can be observed as a change in the color of the reaction agent from violet to yellow and a reduction in the absorbance at 517 nm [28]. The ANOVA demonstrated that the tested extracts showed similar scavenging activities among themselves and statistically significantly lower (*P* < 0.05) from that of *Ginkgo biloba* extract and other standards such as gallic acid, vitamin C and rutin. However, the DPPH scavenging activities of the isolated pure substances were higher and the results do not disqualify the antioxidant activity of the tested samples (Table 3). The results here represent a basis for the quality control of this plant drug, as there are no reference values described in the literature for the bark of *E. uchi*.

Parametric and nonparametric correlation tests were done between total phenols content, total tannins content and antioxidant activity. The results are presented in Table 4.

These results suggest that there was no relationship between the total tannins content in extracts and their antioxidant activity (values close to zero) and that there was a significant negative relationship between total phenols and total tannins and among total phenols and antioxidant activity.

The high antioxidant activity coupled with low cytotoxicity of the extracts, in addition to the previously reported lack of acute oral toxicity [29], and selective anti-inflammatory activity [30], encourage further studies in search of possible potential anti-cancer activities.

## 3. Experimental Section

### 3.1. Plant Material

Powdered bark of *Endopleura uchi* (Huber) (Humiriaceae) was acquired from the Sítio da Mata company (lot: Uxi03/01; N°Inscr. Prod.: 024.308.176; crop: 01/09/2005; expiry: 30/09/2008), located on the Cajuru highway, Cassia dos Coqueiros (SP), Brazil.

### 3.2. Preparation of Plant Extracts

Plant extracts were prepared in the concentrations of 5, 10 and 20% (w/v), by various procedures: (1) maceration (M), (2) turbo-extraction (T), (3) percolation (P), (4) infusion (I), (5) decoction (D). In the first three procedures, 50% ethanol (EtOH 50%) was used as solvent and in the last two, distilled water. The extracts were dried and concentrated under reduced pressure in rotaevaporator at 40 °C, the yields obtained (w/v) were: 4.56–11.54% to 5% extracts; 5.59–14.17% to 10% extracts and 4.37–23.87% to 20% extracts. The extracts were preserved in a desiccator to avoid humidity incorporation.

### 3.3. Total Tannin Content in Plant Extracts

The total tannin content was estimated by a colorimetric assay based on procedures described by Glasl (1983) [31] and Farmacopéia Brasileira IV (1996) [32], with slight modifications. To determine the total tannins of a plant, it is recommended to use a sample of 0.750 g [32]. However, for the extracts, it was necessary to make a small correction [23]. Thus, the amount of plant sample weighed for each extract was calculated as: *M*_extract_ = (%) extractive content of extract (*EC*) × 0.750/100, where EC is the percent of dry residue weight extracted from the sample and *M*_extract_ is the mass of extracts.

Briefly, the samples were dissolved in 250 mL water to give the mother liquor (ML). A 5 mL aliquot of ML was diluted in water to 25 mL and 2 mL of this solution, were transferred to a 25 mL vial with Folin-Ciocalteau phenol reagent 2 N (1 mL) and Milli-Q^®^ water (10 mL) and made up to volume with a 10.6% sodium carbonate solution. After 15 minutes, the absorbance was read at 730 nm. Water was used as the blank. To determine the non-adsorbent polyphenols (NAP), 10 mL ML was mixed with hide powder (Merck^®^) (100 mg) and shacked for 60 minutes. A 2 mL aliquot of this solution was assayed for polyphenolics as above. The absorbance wavelength (730 nm) was previously selected by spectrophotometric scanning of samples of extract and gallic acid. The percentage of total phenolics and tannins were determined as follows: *TP* (%) = [15625 × *Abs*]/[1000 × *m*], *NAP* (%) = [15625 × *Abs*]/[1000 × *m*], *TT* (%) = *TP* − *NAP*, where *TP* = total polyphenolics (%); *NAP* = non-adsorvent polyphenolics (%); *Abs* = Absorbance; *m* = mass (g) of samples; *TT* = total tannins (%).

### 3.4. Antimicrobial Assays

The agar disk diffusion technique (both with “templates” and “paper discs”) was employed to test for antimicrobial activity against strains of *Bacillus subtilis* (ATCC 9372), *Staphylococcus aureus* (ATCC 25923), *Staphylococcus epidermidis* (ATCC 27853), *Escherichia coli* (ATCC 25922), *Shigella sonnei* (clinical isolate) and *Candida albicans* (ATCC 64548). A colony of each bacterial strain used, or 150 μL of a previously prepared bacterial suspension was inoculated in Brain Heart Infusion (BHI) broth and incubated at 37 °C for 24 hours. The turbidity of each bacterial cell suspension was then adjusted with saline to match the 0.5 McFarland scale (∼1.5 × 10^8^ CFU/mL). Five colonies of *Candida albicans* were inoculated in Sabouraud broth and incubated at 35 °C for 24–48 hours. The turbidity of the yeast cell suspension was then adjusted to 0.5 on the McFarland scale (1× 10^6^–5 × 10^6^ CFU/mL) with sterile saline. Ampicillin (50 μg/mL) was used as a positive control for the bacterial strains and amphotericin B for the *Candida albicans* strain. The later was prepared by dissolving 16 mg of amphotericin B in 10 mL of dimethylsulfoxide (DMSO) and diluting this, twice, in the proportion 1:5 (w/v), obtaining the stock solution 64 μg/mL.

The agar diffusion tests were performed as in the approved standards M2-A8 and M44-A of the Clinical and Laboratory Standards Institute, with modifications [33,34]. Bacterial and yeast inoculums were prepared as described previously adjusting the turbidity to the McFarland scale. Müller-Hinton agar (MHA) for bacteria, or MHA supplemented with 2% glucose and 0.5 μg/mL methylene blue for yeast, were poured into sterilized Petri dishes, having been seeded with previously prepared inocula (150 μL of bacterial suspension or 100 μL of yeast suspension). Disk diffusion templates (6 wells of 6 mm internal diameter) or paper discs (6 mm diameter) were placed on the seeded plates. To each well, 50 μL of ampicillin/amphotericin B solution, 50 μL of each *E. uchi* extract (10 mg/mL, resuspended in DMSO) and 50 μL of DMSO:BHI solution (1:1 v/v) as a negative control were added. Sterilized filter paper discs (Whatman^®^ grade No. 1) were individually impregnated with 40 μL of each 10 mg/mL extracts solution (resuspended in DMSO), 40 μL of DMSO:BHI (1:1, v/v) as negative control and 40 μL of ampicillin or amphotericin B as positive controls. The plates were incubated at 37 °C for 24–48 hours. The inhibition of the bacterial and/or fungal growth was determined by measuring the haloes around of the wells and discs with the aid of a digital calliper, and expressed as the average of three independent experimental determinations.

### 3.5. Broth Micro-Dilution Assay for Minimum Inhibitory Concentrations (MIC)

The minimum inhibitory concentration (MIC) of the extracts that presented some activity against the tested microbial strains was determined by the broth micro-dilution method, as described in the M7-A6 reference guideline of the Clinical and Laboratory Standards Institute, with modifications [35].

The test was carried out in BHI broth for bacterial strains or RPMI-1640 (adjusted to pH 7.0 with 3-(*N*-morpholino) propanesulfonic acid (MOPS) buffer, 0.165 mol/L) for the yeast in 96-well flat-bottomed microtitration plates, containing 0.1 mL medium in each well. The extracts were prepared in DMSO at an initial concentration of 200 mg/mL and diluted in DMSO:BHI (1:5, v/v) to obtain 10 mg/mL in test solutions. Sample solutions (0.1 mL) were two-fold serially diluted in the plates with liquid medium. The working inoculum suspension (0.1 mL) was added to give a final inoculum concentration of 1 × 10^5^–5 × 10^5^ and 0.55 × 10^3^–2.5 × 10^3^ CFU/mL, for bacteria and yeast assays, respectively. Ampicillin final dilutions ranging from 12.5 μg/mL to 0.012 μg/mL and amphotericin B final dilutions ranging from 16 μg/mL to 0.015 μg/mL were used for bacteria and yeast, respectively, as positive control. Negative contamination controls using only medium (BHI or RPMI), and with or without extract were used in the tests. The plates were incubated at 37 °C for 24 and 48 hours for bacteria and yeast, respectively. No inhibitory effects were observed in the presence of DMSO at the highest concentration used. To perform the visual reading, 20 μL of 0.01% (w/v) resazurin (Sigma^®^) was added to each well. The plates were incubated for a further 30 minutes, and estimated visually for any change in color from blue to pink, indicating reduction of the dye due to microbial growth. The lowest concentration that remained blue was taken as the MIC. Experiments were performed in duplicate.

### 3.6. Cytotoxicity Assay

Cytotoxicity was tested on rabbit corneal fibroblasts (SIRC, CCL-60). These cells were maintained in culture bottles incubated at 37 °C with 5% CO_2_ in Eagle medium (pH 7) supplemented with 15% fetal bovine serum, without sodium bicarbonate. The extract samples were prepared by dissolving 10 mg of 20% decoction, 10% infusion, 5% maceration (EtOH 50%), 10% percolation (EtOH 50%) and 20% turboextraction (EtOH 50%) in 1 mL of DMSO. These initial stock solutions were diluted (1:5, v/v) in Eagle medium, to obtain the test solutions.

For cytotoxicity evaluation, the cells were collected, centrifuged (1500 rpm, 10 minutes) counted and adjusted to the concentration 1 × 10^5^ cells/mL in Eagle medium. The cells were incubated in 96-well flat-bottomed microtitration plates at 37 °C in an atmosphere of 5% CO_2_ for 72 h. *E. uchi* samples, diluted in DMSO:Eagle (1:10, v/v), were added. The extracts were serially diluted (1:1, v/v) across the plate resulting in an initial concentration of 200 μg/mL to a final concentration of 39 μg/mL. The plate was incubated for 24 hours at 37 °C in a humid atmosphere with 5% CO_2_ [36–38]. After this, 15 μL of resazurin aqueous solution (0.1 mg/mL) was added. The plate was incubated in a humid atmosphere with 5% CO_2_ at 37 °C. The wells were read visually after 3 hours by distinguishing the original blue color (absence of living cells) from pink (presence of living cells) and with a microplate fluorescence reader (Spectra Fluor Plus, Analysis program Magellin), with filters of 530 and 590 nm [39].

### 3.7. Scavenging Activity of DPPH Radical

The assay of antioxidant activity of the extracts was based on the scavenging activity of 2.2-diphenyl-1-picrylhydrazyl solution (DPPH) [40]. Gallic acid, rutin and vitamin C were used at a concentration of 250 μg/mL (in methanol), as antioxidants [41]. A *Ginkgo biloba* L. extract (Santosflora^®^) was also used in this test, at the concentration of 250 μg/mL (in methanol), for its recognized antioxidant activity [42]. The tests were performed on the extracts: 20% decoction; 10% infusion; 5% maceration (EtOH 50%); 10% percolation (EtOH 50%) and 20% turboextraction (EtOH 50%). To 1 mL of the sample solutions, 2.5 mL of 0.004% DPPH in methanol solution were added. The resulting solutions were homogenized by vortex and kept in the dark for 30 minutes at room temperature. A solution of DPPH in methanol (2.5:1, v/v) was used as negative control, and methanol was used as blank. Absorbance was measured at 517 nm in a Shimadzu-1603 spectrophotometer, with quartz cuvettes of 1 cm light pathway. The anti-radical activity was calculated by the equation: percent radical scavenging activity = (*Abs*_control_ − *Abs*_sample_)/*Abs*_control_ × 100, where *Abs* = absorbance.

### 3.8. Statistical Analysis

The results were expressed as the average of three determinations ± standard deviation, using Microsoft Office Excel 2007^®^. When necessary, analysis of variance (ANOVA) was performed, with *P* < 0.05. The Spearman and Kendall-Tau tests (*α* = 0.05) were performed with Stat Plus Professional 2009^®^ to verify correlation coefficient among total tannins content, total phenols content and antioxidant activity.

## 4. Conclusions

Although satisfactory antimicrobial activity was not observed for any extract used, new tests with more sensitive techniques should be performed, since high levels of tannins were found in the samples, which are well recognized for antimicrobial properties. Moreover, the absence of cytotoxicity and high antioxidant activity and the previously reported lack of acute oral toxicity, confirm that the bark used in the preparation of teas by the population can be consumed safely. The results presented here, in association with others in the literature, should guide future work in the search for a possible anti-cancer activity.

## Figures and Tables

**Table 1.** Extractive content and total tannins content for each extraction method.

	**Decoction**	**Infusion**	**Maceration**	**Percolation**	**T. Extraction**
**EC_5%_**	12.53 ± 1.02	9.7 ± 0.05	13.73 ± 0.11	18.6 ± 0.26	12.83 ± 0.32
**TT_5%_**	19.65 ± 1.59	22.66 ± 0.53	23.62 ± 1.00	20.72 ± 0.79	19.99 ± 0.49
**EC_10%_**	11.41 ± 0.40	8.85 ± 0.05	13.1 ± 0.17	16.8 ± 0.39	12.95 ± 0.62
**TT_10%_**	16.34 ± 3.18	26.06 ± 4.27	23.41 ± 2.47	32.85 ± 1.62	20.26 ± 0.50
**EC_20%_**	9.65 ± 0.22	8.525 ± 0.25	13.04 ± 0.05	18.87 ± 0.27	14.85 ± 0.54
**TT_20%_**	20.27 ± 1.16	12.08 ± 2.11	23.18 ± 0.93	18.56 ± 1.79	24.77 ± 2.54

EC_5%_: extractive content of 5% extracts; TT_5%_: total tannins of 5% extracts; EC_10%_: extractive content of 10% extracts; TT_10%_: total tannins of 10% extracts; EC_20%_: extractive content of 20% extracts; TT_20%_: total tannins of 20% extracts.

**Table 2.** Diameters of the haloes (mm) of bark extracts of *E. uchi* in the agar diffusion test performed with templates and with filter paper discs.

	**Antimicrobial Assays**					
	**Template Technique**					

**Samples**	**SA**	**EC**	**BS**	**SS**	**SE**	**CA**

**D_20_**	15.0	−	−	−	−	−
**I_10_**	15.0	−	−	−	−	−
**M_5_**	13.0	−	−	−	−	−
**P_10_**	13.0	−	−	−	−	−
**T_20_**	14.0	−	−	−	−	−

**A**	23.0	18.0	15.5	13.0	17.5	23.5
**C**	−	−	−	−	−	−

	**Paper Disc Technique**					

**Samples**	**SA**	**EC**	**BS**	**SS**	**SE**	**CA**

**D_20_**	7.0	−	−	−	−	−
**I_10_**	7.0	−	−	−	−	8.0
**M_5_**	7.0	−	−	−	−	−
**P_10_**	8.1	−	−	−	−	−
**T_20_**	7.9	−	−	−	−	−

**A**	19.3	13.7	19.7	17.5	12.7	17.5
**C**	−	−	−	−	−	−

D_20_: 20% decoction (w/v); I_10_: 10% infusion (w/v); M_5_: 5% maceration (w/v); P_10_: 10% percolation (w/v); T_20_: 20% turboextraction (w/v); A: antibiotic, C: negative control, SA: *Staphylococcus aureus*; EC: *Escherichia coli*; BS: *Bacillus subtilis*; SS: *Shigella sonnei*; SE: *Staphylococcus epidermidis*; CA: *Candida albicans*.

**Table 3.** Radical-scavenging activity of standards and sample extracts (250 μg/mL).

**Samples (250** μ**g/mL)**	**Radical Scavenging Activity (%)**
Gallic acid	97.24 ± 0.05
Rutin	96.83 ± 0.06
Vitamin C	98.14 ± 0.06
*Gingko biloba* extract	96.97 ±0.09
D_20_	87.46 ± 2.59
I_10_	79.54 ± 2.63
M_5_	89.22 ± 0.82
P_10_	88.90 ± 2.52
T_20_	90.58 ± 0.62

D_20_: 20% decoction (w/v); I_10_: 10% infusion (w/v); M_5_: 5% maceration (w/v); P_10_: 10% percolation (w/v); T_20_: 20% turboextraction (w/v).

**Table 4.** Parametric and non parametric tests for correlation among total tannins content, total polyphenols content and antioxidant activity.

	**Spearman Test (***α***= 0.05)**	**Kendall-Tau Test (***α***= 0.05)**	**Pearson Test (***α***= 0.05)**
TPC-TTC	*r* = −0.7; p-level = 0.18	*t* = −0.6; p-level = 1.00	*ρ* = −0.6636; p-level = 0.22
TTC-AOA	*r* = −0.1; p-level = 0.87	*t* = 0; p-level = 1.00	*ρ* = −0.0065; p-level = 0.99
TPC-AOA	*r* = −0.6; p-level = 0.28	*t* = −0.4; p-level = 0.32	*ρ* = −0.1937; p-level = 0.75

PTC: total polyphenols content; TTC: total tannins content; AOA: antioxidant activity; *r*: Spearman correlation coefficient; *t*: Kendall-Tau correlation coefficient; *ρ*: Pearson correlation coefficient.
